# Development of spinal deformities in the tight-skin mouse

**DOI:** 10.1038/boneres.2016.53

**Published:** 2017-02-21

**Authors:** Bing Li, Jill PG Urban, Jing Yu

**Affiliations:** 1 Department of Orthopedics, Tianjin Hospital, Tianjin, China; 2 Department of Physiology, Anatomy and Genetics, University of Oxford, Oxford, UK

## Abstract

Tight-skin (TSK) mice are commonly used as an animal model to study the pathogenesis of Marfan syndrome (MFS), but little is known of their skeletal phenotype and in particular of the development of the spinal deformities, common in MFS. Here we examined growth of the axial skeletons of TSK and wild-type(B6) mice during their period of rapid growth. The whole bodies of mice, 4–12 weeks of age, were scanned after sacrifice, by micro-computed tomography (microCT). We reconstructed three-dimensional models of the spine and ribs, and measured vertebral body heights and rib lengths using the Mac-based image-processing software “OsiriX”. Although the TSK mice were smaller than the B6 mice at 4 weeks, they experienced an early growth spurt and by 8 weeks the height, but not the width, of the vertebral body was significantly greater in the TSK mice than the B6 mice. Measurement of the angles of scoliotic and kyphotic curves post-mortem in the mice was problematic, hence we measured changes that develop in skeletal elements in these disorders. As a marker of kyphosis, we measured anterior wedging of the vertebral bodies; as a marker for scoliosis we measured asymmetries in rib length. We found, unlike in the B6 mice where the pattern was diffuse, wedging in TSK mice was directly related to spinal level and peaked steeply at the thoracolumbar junction. There was also significant asymmetry in length of the ribs in the TSK mice, but not in the B6 mice. The TSK mice thus appear to exhibit spinal deformities seen in MFS and could be a useful model for gaining understanding of the mechanisms of development of scoliosis and kyphosis in this disorder.

## Introduction

Deformities of the spinal column, such as scoliosis, which develop during growth are relatively common. Mild scoliotic curves, resulting in poorer health, are evident in 6% of the population;^1^ around 5% of these curves progress to the stage where intervention is required^2^ with the medical costs of surgical curve correction being over $1 billion per year in the USA alone.^3^ Although in most cases, the causes of scoliosis are not well understood and are thought to be complex and multifactorial,^4^ in other cases, such as in Marfan syndrome (MFS), scoliosis can arise from a mutation of a single gene.

MFS is a autosomal dominant disorder caused by mutations in the gene encoding fibrillin-1.^5,6^ Patients with MFS have a defect in the synthesis, secretion, or incorporation of fibrillin-1, a glycoprotein that is a major component of various types of connective tissues, including those of the skeleton.^7^ Although the ocular and cardiovascular systems have been investigated intensively, skeletal manifestations that range from disproportionately long limbs to scoliosis,^8–11^ have been less studied; scoliosis is a particular problem in MFS with a prevalence of 60%;^9^ around one third of these scoliosis cases require surgical correction.^10,11^

Tight-skin (TSK) mice are commonly used as an animal model to study the pathogenesis of MFS. TSK is an autosomal dominant mutation discovered in 1976 at The Jackson Laboratory and first described by Green *et al.*^12^ The mutation, located on mouse chromosome 2, is associated with an intragenic duplication of the fibrillin (*Fbn 1*) gene and has a generalized effect on connective tissues. The phenotype overlaps with that of MFS and has been widely studied as a paradigm for scleroderma,^13^ emphysema,^14^ and cardiovascular disease.^15^ TSK mice also exhibit the skeletal manifestations of excessive growth in limbs and in regions of the axial skeletons^12,16^ seen in MFS. However, little is known of the whether the TSK mice develop the spinal deformity seen in MFS patients.

The purpose of this study was to investigate whether a measurable spinal deformity developed in TSK mice. MicroCT images of the spines of TKS mice and their wild-type (WT) controls (B6 mice) were obtained every 2 weeks at 4–12 weeks and 3D images of the spines were constructed using OsiriX software to enable measurement of the vertebral bodies and ribs. Although initially smaller than the B6 mice, from 4 weeks of age, the spinal elements of the TSK mice grew more rapidly than those of the B6 mice. The TSK mice also showed significant asymmetry in rib length between left and right ribs in the thoracic spine, whereas vertebral body asymmetry (posterior versus anterior height) was particularly marked at the thoracolumbar junction. These differences in growth rates and in the development of rib asymmetry and vertebral wedging are similar to those seen in scoliotic and kyphotic patients. These mice could thus provide a model system for investigations into how a single gene mutation could lead to the development the spinal deformities seen in MFS patients.

## Materials and methods

### Animals

All procedures were reviewed and approved by the Institutional Animal Care and Use committee of University of Oxford. 15 C57BL/6J (B6) WT mice and 27B6.Cg-*Fn1*^*Tsk*^*+/+Bloc1s6*^*pa*^*/J*(TSK)mice (all female and 2 weeks of age), were purchased from The Jackson Laboratory (JAX), Bar Harbor, USA. Mice were examined between 4 and 12 weeks to cover periods of rapid growth and puberty.^17^ Each mouse was assigned to one of the 5 groups: 4 weeks (*n*=3 and *n*=6 for B6 and TSK, respectively), 6 weeks (*n*=3 and *n*=6), 8 weeks (*n*=3 and *n*=5), 10 weeks (*n*=3 and *n*=5), and 12 weeks (*n*=3 and *n*=5). Each group of mice was weighed and killed at the relevant age and then stored in −20 °C until scanned by microCT.

### MicroCT and image processing

The microCT images were obtained at the Radiology Research Institute, University of Oxford with the help of Dr. Sean Smart and Dr Kersekans-Veele. A Siemens microCT (Siemens Inveon PET-CT scanner) was used to scan the whole body of the mice excluding the tail, at a resolution of 50 μm using a dedicated cradle and holder. Scans were performed at 360°, involving 360 steps and continuous rotation. (Scanning details: (i) 3 072×2 048 FOV using binning of 4 (ii) exposure time was 400 ms (iii) 1 bed position, low magnification (resulting in a detector pitch of 104 μm) (iv) tube at 65 kV and 500 μA).

All the cross-sectional raw images collected by microCT were transferred into DICOM format by the public domain, Java-based processing software “Image J” (Mac OS X version; “imagej.nih.gov.ij/download.html”).

### Measurements of vertebral bodies and ribs

**Figure 1:**
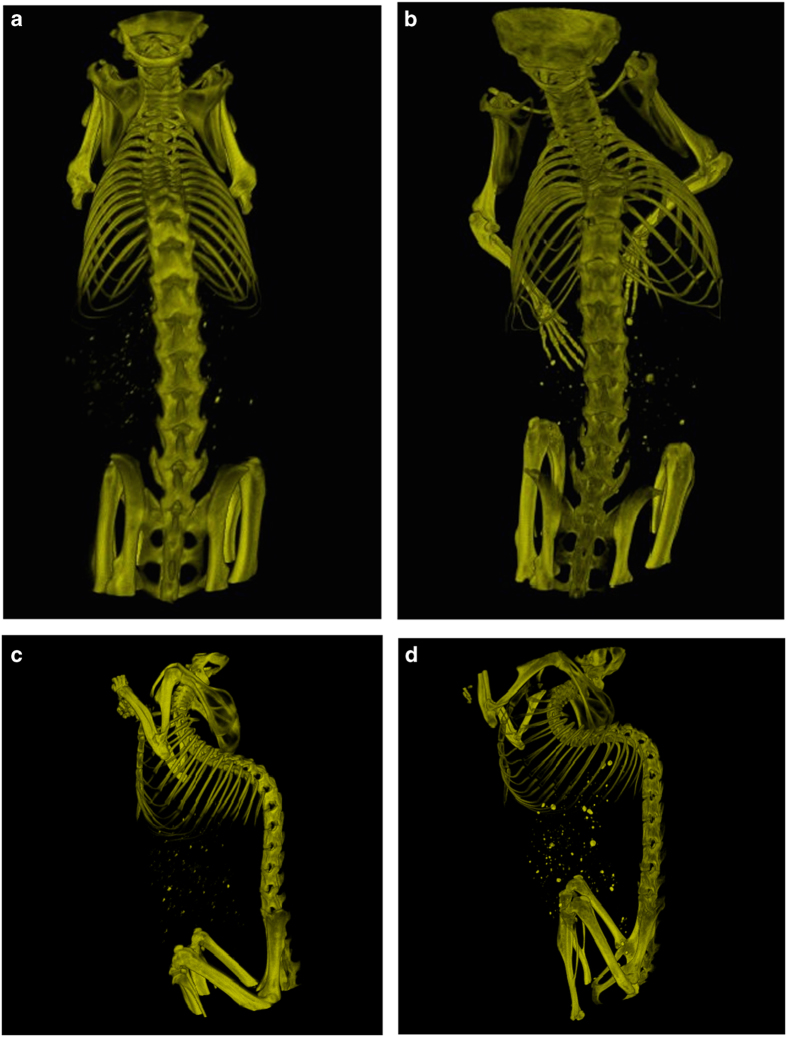
Typical 3D reconstructions of microCT scans at 12 weeks of age. These show AP views (**a**,** b**) and lateral views (**c**, **d**) of B6 mice (**a**,** c**) and TSK mice (**b**,** d**).

**Figure 2:**
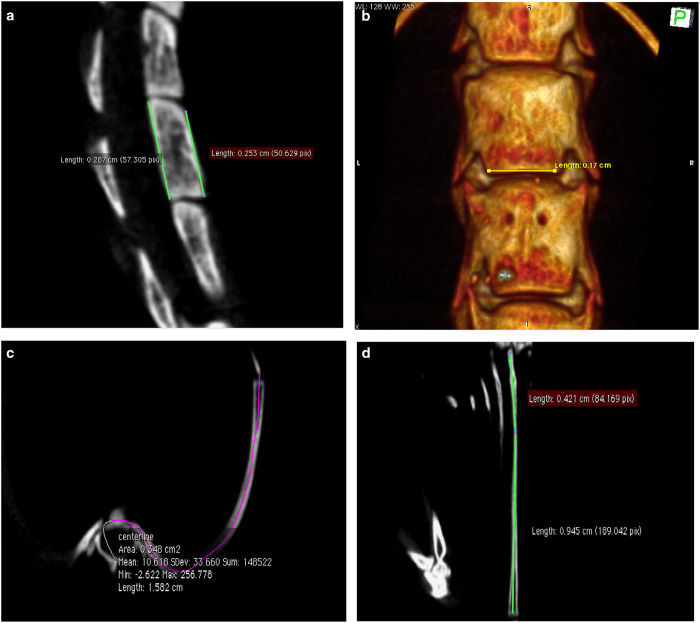
Measurement of vertebral body height and width and of rid length using “OsiriX”. The linear measurement tool in “OsiriX” was used to for both width and height measurements. (**a**) The anterior and posterior heights of the vertebral body, ranging from T4 to L6, were measured on the median sagittal view of the vertebral body (green lines). (**b**) The inferior vertebral body width, ranging from thoracic vertebra 4 to lumbar vertebra 6 (T4–L6), was measured in frontal view of the 3D image, reconstructed by “OsiriX”. The 3D spine model was roatated and cut out find the appropriate position, then a line was drawn on the bottom of the vertebral body for width measurement, as shown (white arrow) (**c**) shown the centerline (purple) along the rib from the sternocostal synchondrosis to the end of rib. (**d**) The measurement of rib length (two green lines) using the linear measurement tool according to the CPR reconstruction. The sum length of the two green lines equals the rib length.

A Mac system based software “Osirix” (32 bit, Pixmeo SARL, Switzerland) was used to perform 3D reconstructions of DICOM images (Figure 1) and all the associated measurements. Measurements were carried out from T4 to L6 as the region of interest in development of vertebral deformities. The linear measurement tool in Osirix was used to measure length of the anterior and posterior vertebral bodies (Figure 2a) and vertebral body width (Figure 2b). As the rib is curved in three-dimensional space, the linear measurement tool cannot be used for its length measurement. The Curve planar reformation (CPR) function in “OsiriX” was used to measure the rib length. Briefly, a “centre line” was drawn along the rib between the tip of costal head and the osseous end of the rib through the continuous cross-sectional images (Figure 2c). Then the rib length was measured using the linear measurement tool according to the 2D image reconstructed by CPR, in which the 3D curved rib was stretched. If the rib was not completely straight after reconstruction, multi-lines were used for rib measurement (Figure 2d).

### Statistical analysis

Data are presented as mean±s.d. or ratios as appropriate. An unpaired *T-*test was used to compare the mean value of body weight, scoliosis angle, kyphosis angle, vertebral body width and height, and of rib length between TSK and B6 mice at each time point. The *χ*^2^-test was performed to test whether the number of asymmetric ribs in the TSK mice compared with the WT mice was different to that expected (by assuming that 50% of the ribs are left>right, and 50% would be right>left in length if differences within each mouse were purely due to chance). One-way analysis of variance followed by a Tukey’s *post hoc* were used to compare the growth of ribs and vertebral bodies at different time points within each strain of mice. All the statistical analyses were performed by SPSS for Macintosh, Version 19 (IBM Corp., Armonk, NY, USA), and measurements with *P*<0.05 were considered to be statistically significant. Statistical results are shown in the Supplementary Data.

## Results

### Body weight

The weight of TSK and B6 mice at different times is showed in Table 1. An unpaired *T-*test showed no statistical difference between TSK mice and B6 mice at the beginning of growth (4–6 weeks). After 6 weeks of age, the TSK mice were heavier than B6 mice (*P*<0.05). This increase in weight did not appear to arise from a differences in diet or activity between the two mouse strains.

### Vertebral body heights

#### Change in vertebral body heights for B6 and TSK mice with age and spinal level

**Figure 3:**
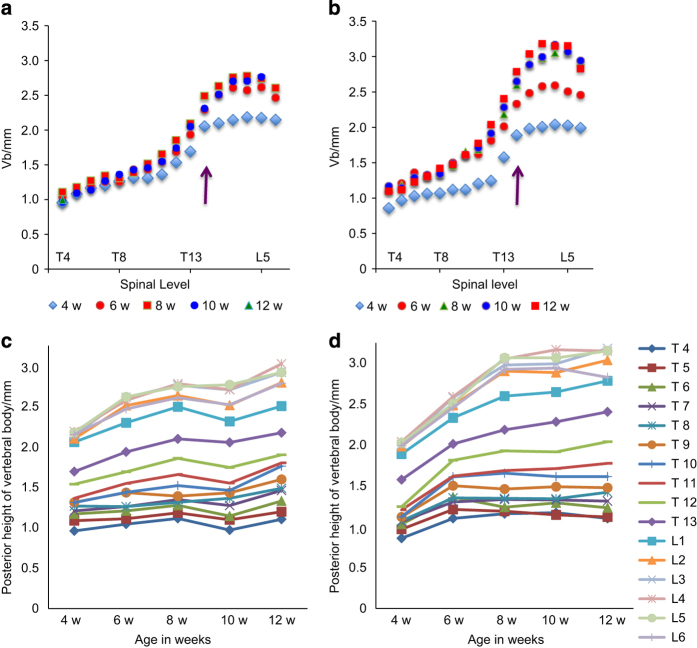
Posterior vertebral body height versus spinal level and age for B6 and TSK mice. Posterior height versus spinal level at different ages (**a**, **b**; purple arrow shows the thoracolumbar junction) and versus age at different spinal levels (**c**, **d**), shown for B6 (**a**, **c**) and TSK (**b**, **d**) mice. The vertebrae of the TSK mice appear shorter than those of the B6 mice at 4 weeks, but then appear to grow more rapidly than those of the B6 mice and by 12 weeks are noticeably longer than the B6 mice particularly in the lumbar region (statistical analysis shown in Supplementary Data).

The measurement of posterior vertebral body height versus spinal level is shown for the B6 mice (Figure 3a) and the TSK mice (Figure 3b) at different ages (4, 6, 8, 10 and 12 weeks). The same data, shown as vertebral body height versus age, at different spinal levels, are shown for B6 (Figure 3c) and TSK (Figure 3d) mice to enable visualization of the changes with spinal level.

At 4 weeks, the posterior vertebral heights of the TSK mice was less than that of the B6 mice at all levels (Figure 3a and c) and the difference was significant for the thoracic vertebrae (Supplementary Figure 1). Vertebrae at the lower thoracic and in the lumbar regions of both B6 and TSK mice increased markedly in height from 4 to 8 weeks (Figure 3a and b), but the increase was markedly steeper in the TSK mice (Figure 3c and d). By 8–10 weeks, the height of the lumbar vertebrae in TSK mice was significantly greater than those of the B6 mice (Figure 3c and d; Supplementary Figure 1). There was a differential rate of growth along the spinal column. In the TSK mice, the height of the thoracic vertebrae increased around 20% from 4 weeks to 12 weeks of age, that of the lumbar vertebrae increased around 50%, whereas the greatest increase in growth was seen for the vertebrae at the thoracolumbar junction, which increased in height by 55%–65%. In the B6 mice, the height of the thoracic vertebrae increased around 10% between 4 and 12 weeks, that of the lumbar vertebrae increased by 20%–25% and no peak in growth rate was apparent at the thoracolumbar junction. A similar pattern was seen for growth in height of the anterior vertebrae in relation to age and spinal level, and differences between TSK and B6 mice (not shown).

**Figure 4:**
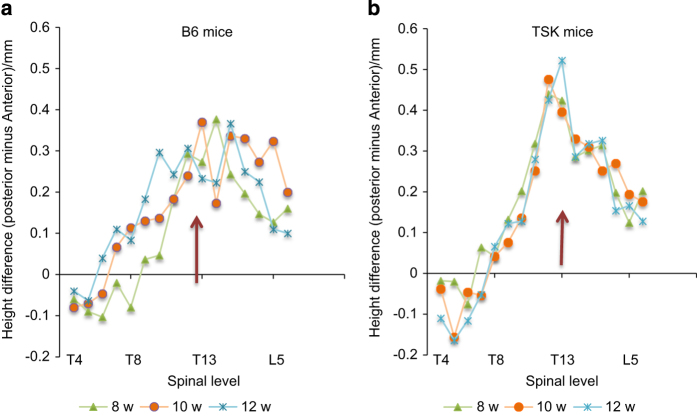
Asymmetry in vertebral body heights versus spinal level. The difference between the posterior and anterior vertebral body heights at different spinal levels is shown for B6 (**a**) and TSK mice (**b**) at 8, 10, and 12 weeks. Thoracolumbar junction (T13–L1) is shown by a purple arrow. The posterior height is greater than the anterior height for all vertebrae from mid-thoracic to lower lumbar for B6 mice, with no clear pattern with spinal level or age. For TSK mice, there is a steep increase in asymmetry with spinal level in the thoracic region which peaks at the thoracolumbar junction, and then decreases steeply towards the lower lumbar levels.

The difference between posterior and anterior vertebral heights in relation to spinal level is shown for B6 (Figure 4a) and Tsk (Figure 4b) mice at 8, 10 and 12 weeks. Asymmetry was evident even at 4 and 6 weeks (not shown) but settled to a pattern by 8 weeks for the TSK but not the B6 mice. At all ages examined, for both B6 and TSK mice, the anterior height was greater than the posterior height for the upper thoracic regions. At the lower thoracic and in the lumbar levels; however, the asymmetry was reversed at all times, with the posterior height greater than that of the anterior for both B6 and TSK mice. For B6 mice, however, there was here no consistent pattern of asymmetry with age or spinal level (Figure 4a). However for the TSK mice, the asymmetry increased steeply with level in the thoracic region, peaked at the thoracolumbar junction and then decreased steeply, with the asymmetry remaining constant from 8 to 12 weeks of age (Figure 4b), after the peak growth spurt (Figure 3d).

#### Comparison of vertebral body width between TSK and B6 mice

**Figure 5:**
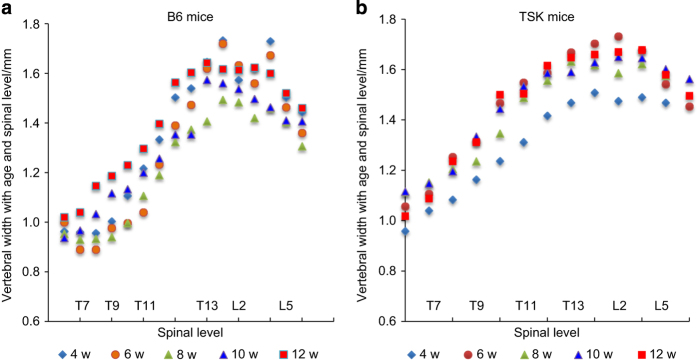
Vertebral width with age and spinal level. This figure shows the width of the vertebrae of B6 (**a**) and TSK (**b**) mice at different ages. The width of the vertebrae increases with spinal level, appears maximal in the lower lumbar levels, and decreases towards the lower lumbar levels in both B6 and TSK mice. There was no apparent increase with age in the B6 mice, however, for the TSK mice, the vertebrae at all levels were noticeably narrower at 4 weeks than at later weeks. No other systematic difference in width with age, or between B6 and TSK mice was detected.

Figure 5 shows the change in vertebral body width with age and spinal level. There was little consistent difference between the width of the B6 and TSK mice at any age or spinal level (Supplementary Data Figure 2). The width of the vertebrae increased with increase in spinal level over the thoracic levels but remained more constant over the lumbar levels. Width increased with age, but less rapidly than the increase in vertebral body height.

### Rib lengths in B6 and TSK mice

**Figure 6:**
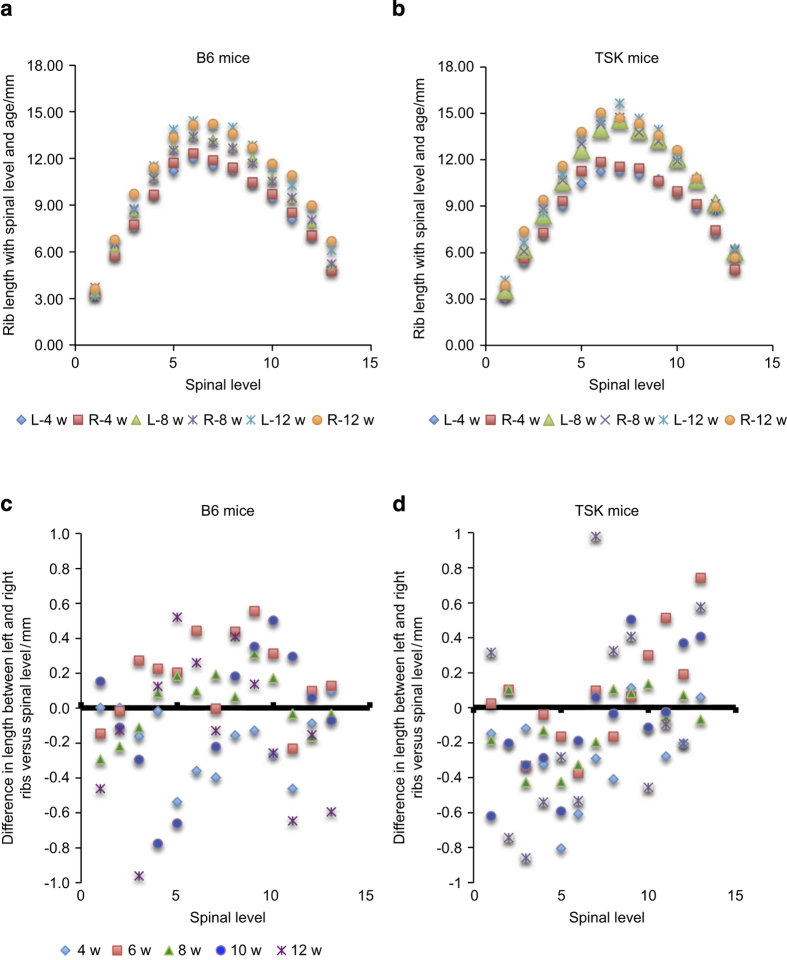
Rib length and rib asymmetry versus age and spinal level. The rib length at different spinal levels (thoracic vertebrae T1–T13) and ages for B6 mice (**a**) and TSK mice (**b**) for both right and left ribs. The difference in length between left and right ribs is shown in (**c**) (B6) and (**d**) (TSK) mice; a negative value is found when the left rib was shorter than the right rib. There is no apparent systematic asymmetry in the B6 mice. For the TSK mice, however, at all ages, the upper thoracic rib lengths were significantly asymmetrical (*χ*^2^ *P*=0.03) with the right ribs longer than the left. Although there was a tendency for a reverse asymmetery in the lower thoracic vertebrae, with the left ribs longer than the right, this difference did not reach levels of significance.

Figure 6 shows left and right rib lengths at different spinal levels and ages for B6 (Figure 6a) and TSK (Figure 6b) mice. Rib length differs markedly with spinal level, being greatest in the mid thoracic region; length here increased markedly between 4 and 8 weeks for the TSK mice (Figure 6c); at 12 weeks the ribs length in the mid-thoracic regions were significantly greater than for the B6 mice (Supplementary Figure 3). Rib asymmetry is shown for B6 (Figure 6c) and TSK (Figure 6d) mice as the mean difference in rib length between the left and right ribs at each level and age; negative values result if the left rib was shorter than the right rib. Although there was no evident systematic asymmetry for the B6 mice, for the TSK mice, the upper thoracic ribs were asymmetric (Figure 6d), with the left rib significantly longer than the right rib at all ages (*χ*^2^ *P*=0.03); an opposite asymmetry is apparent for the lower thoracic vertebrae but did not reach level of significance (Figure 6d).

## Discussion

Here in order to determine whether TSK mice develop the spinal deformities seen in MFS, we examined the vertebral bodies and ribs in TSK mice and their WT (B6) controls from 4 to 12 weeks of age. We found that in both mouse strains, the vertebral body height increased markedly with spinal level, with the heights of the lower lumbar vertebrae more than double those at the upper thoracic levels (Figure 3); these differences in height were maintained over the period of observation. Rib length also varied markedly with spinal level, being greatest around the mid-thoracic levels in both mice strains (Figure 6). However, there were also significant differences in the sizes of the mice themselves and in heights of lumbar vertebrae themselves between the mouse strains. At 4 weeks of age, the TSK mice were lighter than the B6 mice (Table 1) and their the vertebral bodies (Figure 3) and ribs (Figure 6) were shorter those of the B6 mice. From 6 weeks onwards, the TSK mice were significantly heavier than the B6 mice. Also, the height of the vertebral bodies of the TSK mice grew more rapidly than those of the B6 mice with growth rate faster in the lumbar than in the thoracic region; most of the lumbar TSK vertebrae were significantly longer than the B6 mice by 10 weeks (Figure 3, Supplementary Figure S1). There were also differences in rib length between the strains, with changes with age, particularly in the mid-thoracic region more evident in the TSK mice (Figure 6b) than in the B6 mice (Figure 6a). The results indicate an early growth spurt for the TSK mice relative to the B6 mice both in relation to body weight and in height, similar to the early growth spurt seen in Marfan patients relative to the general population.^18^

As well as the phenotype of skeletal overgrowth in MFS, kyphosis, and scoliosis are typical spinal deformities with a prevalence of 60% and 40%,^9,19^ respectively. Whether these skeletal deformities also develop in TSK mice has not been reported, although these mice appear to develop a pronounced hump.^12,16^ We found that quantitative measurement of the deformities in the mice measured post-mortem was problematic because of postural changes after death, probably due to changes in muscle tone.^20^ Hence, as markers of kyphotic and scoliotic deformities, we measured the asymmetries in bone lengths reported to occur in these human spinal deformities.

Anterior wedging of the vertebral bodies is seen in juvenile kyphosis (Scheurmann’s disease); we thus measured the difference between posterior and anterior vertebral body heights as a marker for kyphosis. We found differences between posterior and anterior vertebral body heights (Figure 4a and b), which tended to increase with age between 4 and 8 weeks (not shown). By 8 weeks, in both TSK and B6 mice, the height of the anterior vertebral bodies was greater than the posterior in the upper thoracic levels, but towards the thoracolumbar region, the asymmetry reversed. For the B6 mice there was no consistent asymmetry with spinal level in the thoracolumbar and lumbar region. However, for the TSK mice, here, the change in degree of asymmetry with level was very striking; asymmetry was lowest at the lower thoracic levels, increased markedly towards the thoracolumbar junction, and then decreased steeply in the lumbar levels (Figure 4b) with very similar degrees of asymmetry over the period of 8–12 weeks.

Rib length discrepancy is seen in human scoliotic patients with ribs on the concave side of the curve significantly longer than those on the convex side.^21–23^ Moreover, unilateral shortening or lengthening ribs can induce progressive scoliosis in humans^24^ and in animal models of scoliosis.^25,26^ We thus measured rib length asymmetries as a marker for scoliosis in the TSK and B6 mice. We found significant differences between left and right ribs in the thoracic region of the TSK mice (Figure 6d) with the left ribs shorter than the right ribs, suggesting a curve with the concavity on the right side. At the thoracolumbar region, the asymmetry appeared to reverse, with left ribs tending to be longer than the right ribs though not reaching a level of significance, perhaps indicating development of a double curve in these mice. No significant change asymmetry with spinal level was seen in the B6 mice (Figure 6c).

Hence, the TSK mice but not the B6 mice exhibit many of the pheonotypic skeletal features seen in MFS.^7,19^ The TSK mice show an early growth spurt (Figure 3); in MFS the puberty-associated peak in growth velocity was c. 2 years earlier than in the general population.^18^ They exhibit skeletal overgrowth, a typical feature of MFS; although here it was only measured in the relation to vertebrae, long limbs have been reported in other studies.^16^ As well, the TSK mice exhibit the asymmetries in found in human kyphotic and scoliotic patients (Figures 4 and 6), indicating they develop a kyphoscoliotic deformity.

Although the TSK mouse appears to exhibit the spinal deformities seen in MFS, how the mutation in fibrillin^27^ leads to skeletal overgrowth, differential rates of growth along the spinal column and development of a kyphoscoliosis is unknown. Longitudinal growth of long bones and the vertebrae is governed by activity of the cells of the cartilaginous growth plate; these are embedded in an extracellular matrix containing an extensive fibrillin network.^28^ As fibrillins have a mechanical role and are regulators of growth factor signaling,^29^ the mutations in fibrillin could, as well as altering tissue mechanics, affect signaling of TGF-beta and other growth factors necessary for normal activity of the growth plate^30^ and hence disturb cellular differentiation and the rate of growth. Indeed, asymmetrical changes in cellular and tissue dimensions of growth plates have been seen in animal models of scoliosis^31,32^ and in scoliotic patients.^33,34^ The B6-background TSK mouse thus appears a possible animal model for investigating the mechanisms behind the development of skeletal abnormalities and spinal deformities in MFS, allowing examination of signaling and organization of the growth plates and other spinal tissues, at different spinal levels and at different ages. This information might help in understanding the mechanisms of development of scoliosis and kyphosis in patients with MFS and similar disorders.

## Figures and Tables

**Table 1:** Change in weight of TSK and WT mice during growth

Age/weeks	TSK mice (mean±s.d., g)	WT mice (mean±s.d., g)	*P-*value
4	12.8±2.7	13.8±0.7	0.28
6	16.5±1.8	16.3±0.5	0.40
8	19.6±1.9	17.2±1.1	0.05
10	21.5±0.8	17.2±0.6	0.00
12	22.2±0.6	21.0±1.3	0.04

Abbreviation: TSK, tight skin; WT, wild type.
