# Supplementary material for: Development of spinal deformities in the tight-skin mouse
Source: Bone Res. 2017 Feb 21;5:16053–. doi: 10.1038/boneres.2016.53 (PMC5605766; doi:10.1038/boneres.2016.53)
Supplement: Supplementary Figure 1 [file boneres201653-s1.docx]

FigureS 1a.

|  | T4 | T5 | T6 | T7 | T8 | T9 | T10 | T11 | T12 | T13 | L1 | L2 | L3 | L4 | L5 | L6 |
| --- | --- | --- | --- | --- | --- | --- | --- | --- | --- | --- | --- | --- | --- | --- | --- | --- |
| 4W |  |  |  |  |  |  |  |  |  |  |  |  |  |  |  |  |
| 6W |  |  |  |  |  |  |  |  |  |  |  |  |  |  |  |  |
| 8W |  |  |  |  |  |  |  |  |  |  |  |  |  |  |  |  |
| 10W |  |  |  |  |  |  |  |  |  |  |  |  |  |  |  |  |
| 12W |  |  |  |  |  |  |  |  |  |  |  |  |  |  |  |  |

Pink color indicates statistical significance. W: week; T: thoracic; L: lumbar.

Figure S 1b.

|  | T4 | T5 | T6 | T7 | T8 | T9 | T10 | T11 | T12 | T13 | L1 | L2 | L3 | L4 | L5 | L6 |
| --- | --- | --- | --- | --- | --- | --- | --- | --- | --- | --- | --- | --- | --- | --- | --- | --- |
|  |  |  |  |  |  |  |  |  |  |  |  |  |  |  |  |  |
| 4W |  |  |  |  |  |  |  |  |  |  |  |  |  |  |  |  |
| 6W |  |  |  |  |  |  |  |  |  |  |  |  |  |  |  |  |
| 8W |  |  |  |  |  |  |  |  |  |  |  |  |  |  |  |  |
| 10W |  |  |  |  |  |  |  |  |  |  |  |  |  |  |  |  |
| 12W |  |  |  |  |  |  |  |  |  |  |  |  |  |  |  |  |

Pink color indicates statistical significance. W: week; T: thoracic; L: lumbar.

**Figure S1. Comparison of the vertebral heights of TSK mice with that of B6 mice at different ages**. Figure S1a shows the anterior heights, Figure S1b the posterior heights. At week 4, the anterior and posterior heights of the thoracic vertebrae of the B6 mice were significantly greater than that of the TSK mice. By 10 weeks, the pattern had shifted, and here for most of the lumbar vertebrae, the heights of the TSK mice were significantly greater than those of the B6 mice.
