# Supplementary material for: Development of spinal deformities in the tight-skin mouse
Source: Bone Res. 2017 Feb 21;5:16053–. doi: 10.1038/boneres.2016.53 (PMC5605766; doi:10.1038/boneres.2016.53)
Supplement: Supplementary Figure 2 [file boneres201653-s2.docx]

|  | T4 | T5 | T6 | T7 | T8 | T9 | T10 | T11 | T12 | T13 | L1 | L2 | L3 | L4 | L5 | L6 |
| --- | --- | --- | --- | --- | --- | --- | --- | --- | --- | --- | --- | --- | --- | --- | --- | --- |
| 4W |  |  |  |  |  |  |  |  |  |  |  |  |  |  |  |  |
| 6W |  |  |  |  |  |  |  |  |  |  |  |  |  |  |  |  |
| 8W |  |  |  |  |  |  |  |  |  |  |  |  |  |  |  |  |
| 10W |  |  |  |  |  |  |  |  |  |  |  |  |  |  |  |  |
| 12W |  |  |  |  |  |  |  |  |  |  |  |  |  |  |  |  |

Pink color indicates statistical significance. W: week; T: thoracic; L: lumbar.

**Figure S2. Comparision of vertebral body width between TSK and B6 mice**

At 4 weeks, the lower thoracic and most of the lumbar vertebrae of the B6 mice were statistically wider than those of TSK mice. No consistent significant difference in width was found from 6-12 weeks.
