# Supplementary material for: Development of spinal deformities in the tight-skin mouse
Source: Bone Res. 2017 Feb 21;5:16053–. doi: 10.1038/boneres.2016.53 (PMC5605766; doi:10.1038/boneres.2016.53)
Supplement: Supplementary Figure 3 [file boneres201653-s3.docx]

| Rib  Age | T1 | | T2 | | T3 | | T4 | | T5 | | T6 | | T7 | | T8 | | T9 | | T10 | | T11 | | T12 | | T13 | |
| --- | --- | --- | --- | --- | --- | --- | --- | --- | --- | --- | --- | --- | --- | --- | --- | --- | --- | --- | --- | --- | --- | --- | --- | --- | --- | --- |
|  | L | R | L | R | L | R | L | R | L | R | L | R | L | R | L | R | L | R | L | R | L | R | L | R | L | R |
| 4W |  |  |  |  |  |  |  |  |  |  |  |  |  |  |  |  |  |  |  |  |  |  |  |  |  |  |
| 6W |  |  |  |  |  |  |  |  |  |  |  |  |  |  |  |  |  |  |  |  |  |  |  |  |  |  |
| 8W |  |  |  |  |  |  |  |  |  |  |  |  |  |  |  |  |  |  |  |  |  |  |  |  |  |  |
| 10W |  |  |  |  |  |  |  |  |  |  |  |  |  |  |  |  |  |  |  |  |  |  |  |  |  |  |
| 12W |  |  |  |  |  |  |  |  |  |  |  |  |  |  |  |  |  |  |  |  |  |  |  |  |  |  |

W: week; L: left; R: right; T: thoracic. Pink color indicates statistical difference.

**Figure S3. Comparison of rib length between TSK and B6 mice at different ages and spinal levels.** Fig S3 shows the results of comparison between TSK and B6 mice at 5 different time points. At 4 weeks, only rib 6 on the left side of TSK mice (11.23±0.36, mm) is statistically shorter than that of B6 mice (11.96±0.32, mm). With growth, the number of ribs that are statistically different between TSK and B6 mice increased. Two ribs (1 and 11), seven ribs (from 7 to 13), and all thirteen ribs were found statistically different between TSK and B6 mice at 6, 8 and 10 weeks respectively. Interestingly, the number of statistically different ribs between TSK and B6 mice decreased to 6 when the mice were 12 weeks old.
